# Dermatologic manifestations in an adult with Williams syndrome

**DOI:** 10.1016/j.jdcr.2026.04.031

**Published:** 2026-04-23

**Authors:** Noemi Brigenti, Andrea Danese, Giampiero Girolomoni, Martina Maurelli

**Affiliations:** Section of Dermatology and Venereology, Department of Medicine, University of Verona, Verona, Italy

**Keywords:** advanced glycation end products, onychodystrophy, skin autofluorescence, Williams syndrome

## Introduction

Williams syndrome (WS), also known as Williams-Beuren syndrome, is a rare multisystemic disorder caused by a heterozygous microdeletion at chromosome 7q11.23. Most cases are sporadically, but rare familial transmission has been reported.^1^ The prevalence is estimated approximately 1 in 7500 live births.^2^ The deleted region involves 25-27 genes, with the loss of the elastin (ELN) gene playing a central role in the vascular and connective tissue manifestations.^3^ Molecular basis of WS were identified in the early 1990s,^4^ when fluorescence in situ hybridization studies revealed hemizygosity at the ELN locus. In addition to ELN, other genes contribute to the cardiovascular, metabolic and neurocognitive manifestations of the syndrome through incompletely understood mechanisms.^1^ Diagnosis is usually suspected during childhood based on clinical characteristics and phenotypic features and then confirmed by molecular testing.^1^

Cardiovascular abnormalities represent a defining clinical feature of WS. Supravalvular aortic stenosis and stenosis of the main pulmonary artery branches are the most frequently observed manifestations, although considerable variable severity.^5^ Metabolic comorbidities are also common, particularly insulin resistance, prediabetes, and type-2 diabetes.^1^^,^^5^^,^^6^ Patients also exhibit a recognizable craniofacial phenotype^1^^,^^7^ including increased midface height, broad palpebral fissures, a wide interalar distance, short nasal bridge with reduced nose length and narrow bizygomatic diameter.^8^ Additional distinctive features are the prominent, posteriorly rotated ears with long and narrow conchae, a long philtrum, increased chin height, and full lips with a wide mouth, contributing to the recognizable “*elfin facies.*”^8^ Neurocognitive involvement usually consists of intellectual disability, often associated with relative verbal strengths, along with behavioral features.^1^^,^^2^ Although less studied, a wide range of cutaneous manifestations have been described in patients with WS, including premature graying of the hair, early wrinkling, soft skin texture, and nail abnormalities; these characteristics could provide a relevant clue for identifying WS patients.^7^

## Case report

**Fig 1:**
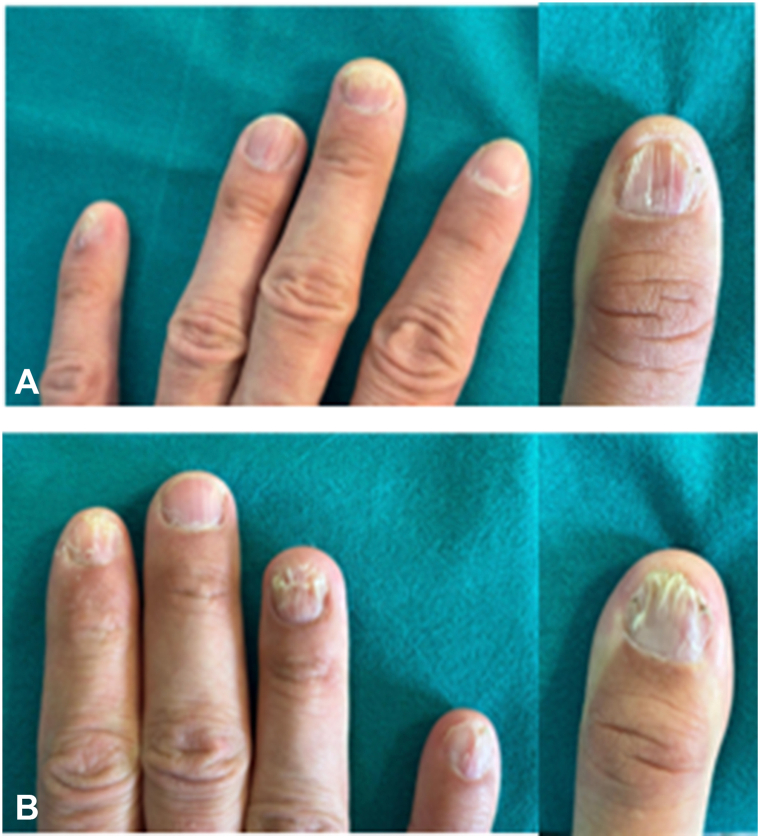
**A,** shows the fingers of the left hand. **B,** shows the fingers of the right hand. Generalized onychodystrophy, bilateral pterygium of the fifth finger, and diffuse ridging and fragmentation can be observed.

**Fig 2:**
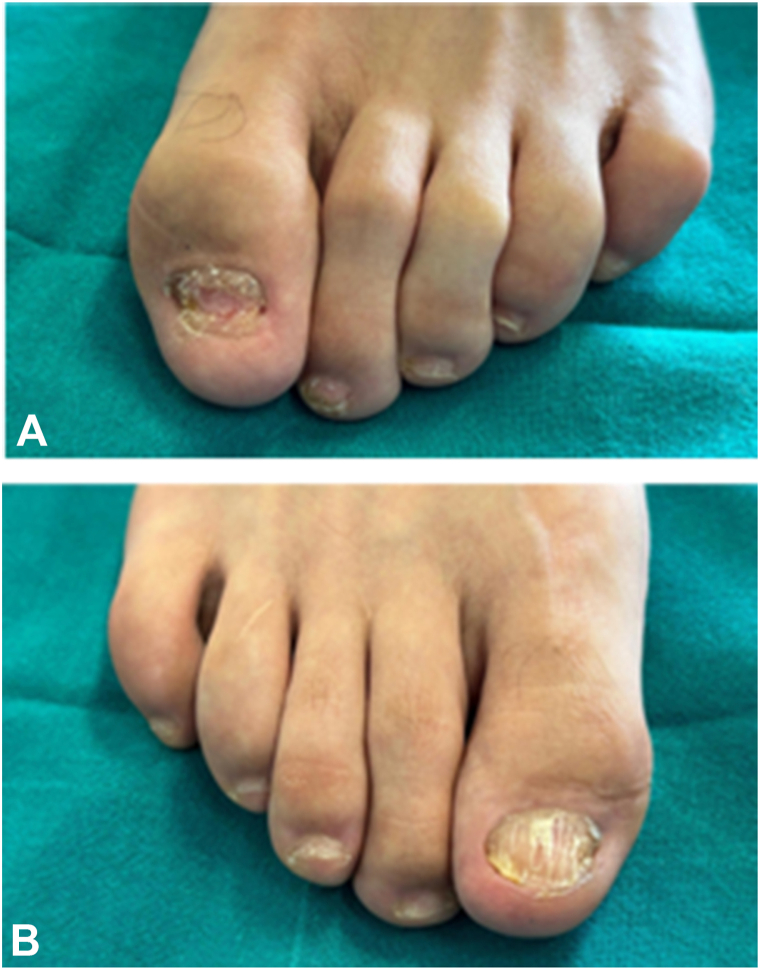
**A,** shows the toes of the left foot. **B,** shows the toes of the right foot; severe onychodystrophy is evident on the hallux of both feet, with particular crumbling of the left hallux; although to a lesser extent, the other toes are also affected by onychodystrophy and distal onycholysis.

**Fig 3:**
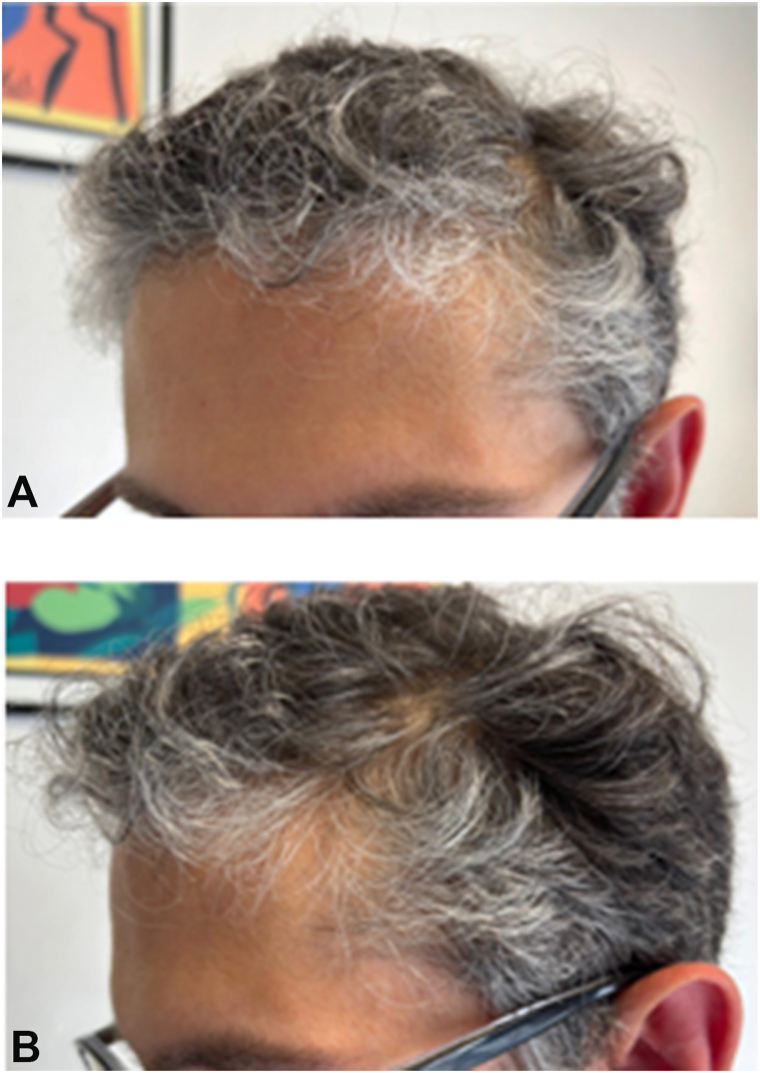
**A, B,** Premature, diffuse greying of the hair, as well as frontal-temporal hairline recession, thinning at the temples, and progressive whitening of the frontal area.

A 28-year-old male patient with WS, confirmed during childhood through fluorescence in situ hybridization test with a specific probe Vysis ELN-gene technology, presented for evaluation of a long-standing nail abnormalities that had worsened over the past year. Clinical examination revealed severe onychodystrophy affecting almost all 20 nails (Figs 1 and 2). Nail findings included longitudinal ridging, onycholysis, and dorsal pterygium in few nails. Additionally, the patient exhibited premature and diffuse graying of the hair, frontal-temporal hairline recession and progressive whitening in the frontal scalp region (Fig 3).

Classic craniofacial traits of WS were observed, including broad forehead (Fig 3), periorbital fullness, malar flattening, long philtrum, full lips, wide mouth (“elfin facies,”) Laboratory tests, including complete blood count, liver enzymes, creatinine, lipid panel, glucose panel, were within normal limits. Because WS is linked to increased metabolic risk,^4^^,^^5^ we performed a non-invasive assessment of Advanced Glycation End Products (AGEs) using the AGE-Reader device (DiagnOptics Technologies B.V., Groningen, The Netherlands).^9^^,^^10^ This technique quantifies tissue AGEs accumulation via skin-autofluorescence and has been validated as a marker of cumulative metabolic and oxidative stress, correlating with cardiometabolic risk.^9^^,^^10^ Results showed an elevated AGEs level (1.80 arbitrary-unit) compared to age-matched reference values (1.56-1.70 arbitrary-unit), suggesting an increased metabolic risk, even though laboratory tests were normal.

## Discussion

Cutaneous manifestations represent an often overlooked component of the WS phenotype. WS is primary known for its cardiovascular and neurocognitive involvement, but several dermatologic features have been consistently reported, reflecting the underlying connective tissue and elastic fiber abnormalities associated with ELN haploinsufficiency.^1^^,^^2^^,^^4^

The WS-Skin and Vessel-Elasticity study provided the most comprehensive characterization of dermatologic findings in WS patients.^7^ In that cohort of 94 patients aged 7-50 years, premature graying of the hair and early facial wrinkling were hallmark features, suggesting an accelerated cutaneous aging process.^8^ Premature hair graying was reported in 58% of participants, sometimes as early as age 11.

Early facial wrinkling was observed in 92% of patients, sometimes starting as early as 7 years, with variable distribution patterns and severity across age groups.^7^ These changes may indicate accelerated aging, by about 3 decades compared to the general population.^7^ Other findings included soft skin (over 80% of cases), abnormal scarring (33%), keratosis pilaris, xerosis or patchy hyperkeratosis, atrophic skin areas and striae distensae. None of these features were present in our patient. Onychodystrophy was noted in 22% of cases and few cases of hypoplastic and ridging nails were reported. Forty-two percent of individuals displayed evidence of, or gave a history of, nail biting, or periungual skin-picking.^7^ Also our patient exhibited several characteristic cutaneous features, including premature diffuse hair graying and severe onychodystrophy involving multiple nails. Common causes of onychodystrophy were ruled out. No other signs to suggest onychomycosis and inflammatory nail conditions such as psoriasis, lichen planus, traumatic, or occupational factors were present and were not confirmed by the patient's clinical history or the results of the physical examination. Without any other systemic or local conditions, nail abnormalities were considered to be part of the spectrum of WS.

This case highlights the skin's role in assessing oxidative stress and cardiometabolic risk in WS. Despite normal routine laboratory findings, skin AGEs revealed increased values compared with age-matched reference values. AGEs are known to be a potential marker of cumulative metabolic and oxidative stress and have been associated with cardiovascular risk and impaired glucose metabolism in various clinical settings.^9^^,^^10^ Patients with WS are at increased risk of cardiometabolic complications, even at a young age.^5^^,^^6^ For this reason, skin AGEs measurement may represent a useful adjunctive tool for risk stratification, potentially allowing earlier identification of individuals at higher risk when standard biochemical markers are still within normal ranges. In clinical practice, measuring skin AGEs could be incorporated as an additional, noninvasive examination in the routine follow-up. This technique is rapid and easy to perform, it does not require specific operator training and is based on a portable device, making it suitable in an outpatient setting.

These findings may support implementation of preventive strategies through dietary and behavioral interventions, as well as pharmacotherapy. These observations highlight the need for structured follow-up protocols for adult WS patients, who may be under-monitored due to their young age. Moreover, there is an unmet need to develop comprehensive, evidence-based protocols tailored specifically for the management of adult WS patients.

## Conflicts of interest

None disclosed.
